# Defining the predictors for post renal transplant left ventricular dysfunction in end‐stage renal disease patients

**DOI:** 10.14814/phy2.70198

**Published:** 2025-02-06

**Authors:** Mahboobeh Sheikhani, Hoorak Poorzand, Mahnaz Ahmadi, Negar Morovatdar, Sara Afshar, Zahra Shahinfar

**Affiliations:** ^1^ Department of Cardiovascular Diseases, Faculty of Medicine Mashhad University of Medical Sciences Mashhad Iran; ^2^ Cardiovascular Division, Vascular and Endovascular Surgery Research Centre Mashhad University of Medical Sciences Mashhad Iran; ^3^ Nephrology Research Center, Montaserieh Hospital Mashhad University of Medical Science Mashhad Iran; ^4^ Clinical Research Development Unit, Imam Reza Hospital Mashhad University of Medical Sciences Mashhad Iran

**Keywords:** CKD, ESKD, LV failure, post renal transplantation, predictors

## Abstract

Reduced left ventricular (LV) function predicts poor outcomes in end‐stage renal disease (ESRD). This study aimed to identify the pre‐renal transplantation echocardiographic parameters that can predict post‐renal transplantation LV failure. This prospective longitudinal study was conducted on patients with ESRD who underwent renal transplantation during 1 year. All patients underwent echocardiography, including ejection fraction (EF), global longitudinal strain (GLS), left ventricular end‐systolic diameter (LVESD), left ventricular end‐diastolic volume (LVEDV), left ventricular end‐diastolic diameter (LVEDD), interventricular septal (IVS) thickness, peak velocity of early diastolic transmitral flow (E), peak velocity of late transmitral flow (A), early diastolic myocardial relaxation (Em), E/A, E/Em, Left atrial volume (LAV) index, tricuspid regurgitation peak gradient (TRPG), systolic pulmonary artery pressure (SPAP), tricuspid annular plane systolic excursion (TAPSE), 1 week before and 1 month after renal transplantation. Fifty patients participated in the current study. All echocardiographic parameters improved after transplantation. Post‐renal transplantation LV dysfunction was observed in 21 (42%) patients. Pre‐renal transplantation echocardiographic parameters (LVEDV, LVESD, LVEDD, IVS, E/Em, TRPG, SPAP, and LAV index) could predict post‐transplantation LV failure with high accuracy (AUC: 0.978).

## 1 INTRODUCTION

Chronic kidney disease (CKD) is an essential public health and economic problem (Lv & Zhang, 2019; Sharma & Sarnak, 2017; Wang et al., 2016). The global prevalence of CKD is 13.4% (Lv & Zhang, 2019). It is estimated that patients with end‐stage renal disease (ESRD) who require renal replacement therapy are between 4.9 and 7.1 million (Lv & Zhang, 2019). The prevalence of ESRD has increased by 70% in 2010 compared to 1990 (Wetmore & Collins, 2016). Based on a meta‐analysis in 2018, the prevalence of CKD in Iran was reported to be 15.14%, which was higher than its global prevalence (Bouya et al., 2018). The prevalence of CKD above grade 4 in heart failure patients was reported to be 10% (Andrassy, 2013; Hein et al., 2019). The prevalence of coronary artery disease and mortality due to myocardial infarction is high among patients with a history of prolonged hemodialysis (Sharma & Sarnak, 2017). The reason for the high prevalence of heart failure in ESRD is due to the presence of renal‐specific risk factors including malnutrition, anemia, uremia, acid–base disturbance, bone mineral disturbances and stunning of the myocardium (Tuegel & Bansal, 2017). ESRD is accompanied by an increased risk of heart failure, cardiovascular mortality, and transplantation failure (Thomas et al., 2017). It was shown that renal transplantation may improve LV hypertrophy and LV function (Chinnappa et al., 2020; Hewing et al., 2016). Regardless of the improvements in LV function after transplantation, cardiovascular disease remains a vital comorbidity in post‐transplant patients (Rao & Coates, 2018). The reasons for cardiovascular disease in post‐renal transplant patients are the persistence of pretransplantation risk factors, including diabetes, dyslipidemia, and hypertension, along with transplantation‐related risk factors, including immunosuppression therapy and patent arteriovenous fistula (Rao & Coates, 2018). Therefore, it is necessary to predict cardiac conditions in post‐renal transplant patients.

Echocardiography is a reliable tool for assessing cardiac function in ESRD (Loutradis et al., 2018). Echocardiography has also been used to predict cardiac function in ESRD patients (Untersteller et al., 2016). Furthermore, pro‐hormone BNP (proBNP) and its non‐active form N‐terminal (NT)‐proBNP are predictors of heart failure in ESRD patients (Bansal et al., 2015; Perez‐Downes et al., 2018; Untersteller et al., 2016). To the best of our knowledge, no comprehensive study has yet assessed the predictors of cardiac conditions, including heart failure in post‐renal transplant patients. Therefore, this study aimed to identify the predictors for heart failure in post‐renal transplantation patients.

## 2 MATERIALS AND METHODS

This prospective longitudinal study was conducted on all patients with ESRD who underwent renal transplantation from September 2019 to September 2020 in a tertiary hospital in Mashhad, Iran. The study was approved by the Ethical Committee of the Mashhad University of Medical Sciences (IR.MUMS.MEDICAL.REC.1397.591). All patients on the renal transplant list of the hospital who were older than 18 years old were included in the study. Exclusion criteria were a history of acute coronary syndrome, cardiac revascularization or wall motion abnormalities in echocardiography, heart failure, severe cardiac valvular disorder, hypertrophic cardiomyopathy, cerebral vascular disease, or peripheral artery disease.

All patients underwent trans‐thoracic echocardiography (TTE) within 1 week before renal transplantation and were followed for 1 month. Conventional TTE and two‐dimensional speckle tracking echocardiography (2D‐STE) studies were performed by the same echocardiologist, using a commercially available EKO 7 diagnostic ultrasound system (Samsung Medison, Seoul, South Korea) with a 2–4 MHz probe, and in accordance with ASE's guidelines (Lang et al., 2015; Nagueh et al., 2016).

All participants were studied with conventional 2‐dimensional echocardiography, pulsed and color Doppler and TDI and the data were analyzed on the machine.

Left ventricular ejection fraction (LVEF) and left ventricular end‐diastolic volume (LVEDV) were measured in the apical four‐chamber and two‐chamber views according to the modified Simpson method. Left ventricular end‐diastolic diameter (LVEDD), left ventricular end‐systolic diameter (LVESD), and interventricular septal thickness (IVS) were measured in the parasternal long‐axis view, using M‐mode. Other echo parameters included Left atrial volume (LAV) index, peak velocity of early diastolic trans‐mitral flow (E), peak velocity of late transmitral flow (A), Mitral annulus velocity on the septal side (Em) obtained by pulsed‐wave tissue Doppler, E/A, E/Em, tricuspid regurgitation peak gradient (TRPG), tricuspid annular plane systolic excursion (TAPSE), and systolic pulmonary artery pressure (SPAP).

LV failure was defined as LVEF of less than 52% for male and 54% for female patients (Lang et al., 2015).

Global Longitudinal strain for left ventricle was measured from the apical four and two chambers and long‐axis view, utilizing STE, on a bull's eye template.

ASE guideline was used for assessment of severity of mitral valve regurgitation, based on not focusing on a single echo parameter (William et al., 2017).

All patients underwent laboratory assessments, including serum creatinine, urea, sodium, potassium, calcium, phosphorus, hemoglobin, hematocrit, and parathyroid hormone (PTH) within 1 week before and 1 month after transplantation. NT‐ProBNP was also assessed before renal transplantation. Systolic and diastolic blood pressure by Vintage mercurial sphygmomanometer, Heart rate and body weight were evaluated before and after transplantation.

### 2.1 Statistical analysis

Data analysis was performed using the Statistical Package for Social Sciences (SPSS) version 16 (IBM Inc. Chicago, Il, USA). The Kolmogorov–Smirnov test was used to assess the normality of continuous. Data were expressed according to the nature of the parametric and non‐parametric disturbance as means ± SD or as number with percentage, respectively. Paired *t*‐test compared the mean of data at baseline with follow‐up data. The Monte Carlo test was used to compare mitral regurgitation severity between baselines and follow‐up. Predictors for LV failure and mitral valve regurgitation were determined using the receiver operating characteristic (ROC) curve. The level of statistical significance was considered as *p* < 0.05.

## 3 RESULTS

Fifty patients (35, 70% males and 15, 30% females) participated in this study. Demographic characteristics of the patients are presented in Table 1. A comparison of laboratory findings of the patients between baseline and follow‐up is presented in Table 2. All laboratory parameters significantly changed after renal transplantation (*p* < 0.001). The mean baseline NT‐ProBNP was 375.06 ± 478.89 pg/mL.

**TABLE 1:** Demographic characteristics of the patients.

Variable	Mean ± SD
Age (years)	33.54 ± 2.45
Weight (Kg)	71.30 ± 8.96
The time between ESRD diagnosis and transplant (years)	5.28 ± 8.96
Heart rate (/min)	80.68 ± 11.99
Systolic blood pressure (mmHg)	139.00 ± 16.24
Diastolic blood pressure (mmHg)	84.76 ± 9.93
Variable	Frequency (%)
Hypertension	26 (52.0%)
Hypertension/hyperlipidemia	6 (12.0%)
Polycystic kidney disease	2 (4.0%)
Hypertension/polycystic kidney disease	1 (2.0%)
Hypertension/Alport	1 (2.0%)
No risk factor	12 (24.0%)
History for minimal CAD	1 (2.0%)

Abbreviations: min, minute, mmHg, millimeter mercury; Kg, kilogram; SD, standard deviation.

**TABLE 2:** Comparison of laboratory findings between baseline and follow‐up.

Variable	Baseline (mean ± SD)	Follow up (mean ± SD)	*p*
Serum urea (mg/dL)	99.80 ± 23.34	31.50 ± 11.05	<0.001*
Serum creatinine (g/dL)	10.47 ± 2.11	1.30 ± 0.19	<0.001*
GFR (mL/min/1.73m^2^)	5.85 ± 1.74	64.61 ± 17.33	<0.001*
Sodium (mg/dL)	141.06 ± 4.27	139.56 ± 3.34	<0.001*
Potassium (mg/dL)	5.38 ± 0.51	4.13 ± 0.36	<0.001*
Phosphorus (mg/dL)	5.10 ± 1.35	2.68 ± 0.52	<0.001*
Calcium (mg/dL)	8.29 ± 0.98	8.9 ± 0.46	<0.001*
Hemoglobin (mg/dL)	11.49 ± 0.97	12.75 ± 0.71	<0.001*
Serum iron (micromole/l)	40.78 ± 22.86	73.78 ± 26.62	<0.001*
TIBC (micromole/l)	389.84 ± 91.16	270.36 ± 41.41	<0.001*
Platelet count (×10^6^/microL)	155.22 ± 35.65	172.54 ± 35.31	<0.001*
Ferritin (ng/mL)	671.00 ± 428.30	205.72 ± 93.00	<0.001*
PTH (pg/mL)	615.72 ± 557.40	88.36 ± 71.02	<0.001*

Abbreviations: GFR, Glomerular filtration rate, mg, milligram, dl, deciliter, g, gram, ml, milliliter, min, minute, m, meter, ng, nanogram, pg, picogram; PTH: parathyroid hormone; SD, Standard deviation; TIBC, Total iron binding capacity

* Significant difference using paired *t*‐test.

A comparison of the echocardiographic parameters of the patients between baseline and follow‐up is presented in Table 3. There was a significant difference between baseline and follow‐up except for A‐wave velocity in echocardiography (*p* = 0.735). LV dysfunction was observed in 21 (42%) patients after renal transplantation. Significant change in MR severity was defined while considering different regurgitation grades, but most included cases in this study had less than moderate regurgitation; So the data in significant MR was not promising. The comparison of study parameters between patients before transplantation based on the presence of LV failure is presented in Table 4.

**TABLE 3:** Comparison of echocardiographic findings between baseline and follow‐up.

Variable		Baseline (mean ± SD)	Follow up (mean ± SD)	*p*
EF (%)		48.30 ± 8.55	52.80 ± 6.86	<0.001*
LVEDD (mm/m^2^)		3.07 ± 0.36	2.91 ± 0.38	<0.001*
LVESD (mm/m^2^)		2.10 ± 0.35	1.94 ± 0.38	<0.001*
LVEDV (mL/m^2^)		70.42 ± 12.70	66.68 ± 10.93	<0.001*
LAV index (mL/m^2^)		27.73 ± 6.98	26.28 ± 6.32	<0.001*
IVS (mm/m^2^)		1.16 ± 0.26	1.02 ± 0.21	<0.001*
GLS (%)		18.06 ± 3.16	20.40 ± 3.21	<0.001*
E (m/s)		75.52 ± 18.5	71.70 ± 16.02	0.021*
A (m/s)		73.6 ± 19.38	72.52 ± 14.73	0.735
E_m_ (m/s)		8.06 ± 1.43	9.08 ± 1.34	<0.001*
E/A		1.12 ± 0.45	1.08 ± 0.27	0.010*
E/E_m_		9.87 ± 3.74	8.19 ± 2.61	<0.001*
TRPG		18.94 ± 8.04	15.54 ± 7.12	<0.001*
SPAP (mmHg)		26.48 ± 11.16	21.84 ± 8.69	<0.001*
TAPSE (mm)		1.83 ± 0.31	1.97 ± 0.28	<0.001*
Variable		Baseline Frequency (%)	Follow up Frequency (%)	*p*
MR severity	No MR	12 (24%)	36 (52%)	<0.001* ^,^ ^a^
	Mild	20 (40%)	15 (30%)	
	Mild to moderate	11 (22%)	8 (16%)	
	Moderate	5 (10%)	1 (2%)	
	Moderate to severe	2 (4%)	0 (0%)	

*Note*: Paired *t*‐test was used for the comparison.

Abbreviations: EF, Ejection fraction; EM, early diastolic myocardial relaxation; IVS, interventricular septal thickness; GLS, global longitudinal strain, LAV, left atrial volume index; LVEDD, left ventricular end‐diastolic dysfunction; LVEDV, left ventricular end‐diastolic volume; LVF, Left ventricular failure; MR: mitral regurgitation; TAPSE, tricuspid annular plane systolic excursion; TRPG, tricuspid regurgitation peak gradient.

^a^ Monte Carlo test was used for the comparison.

* Significant difference.

**TABLE 4:** Comparison of study parameters between patients before transplantation based on the presence of Left ventricular failure.

Variable	Without LVF *n* = 21	With LVF *n* = 29	*p*
Age (years)	28.0 (10.0)	36.0 (14.0)	0.001*
Weight (kg)	72.0 (10.0)	72.0 (15.0)	0.608
Heart rate (/min)	86.0 (17.0)	76.0 (10.0)	0.015*
Systolic blood pressure (mmHg)	125.0 (22.0)	145.0 (13.0)	0.001*
Diastolic blood pressure (mmHg)	80.0 (11.0)	90.0 (7.0)	0.001*
Serum urea (mg/dl)	85.0 (23.0)	96.0 (44.0)	0.013*
Serum creatinine (g/dL)	9.3 (1.9)	10.5 (3.1)	0.047*
GFR (mL/min/1.73m^2^)	6.4 (2.0)	5.0 (2.7)	0.026*
Sodium (mg/dL)	139.0 (5.0)	142.0 (7.0)	0.560
Potassium (mg/dL)	5.3 (0.8)	5.6 (0.5)	0.084
Phosphorus (mg/dL)	4.8 (1.6)	5.0 (1.5)	0.419
Calcium (mg/dL)	8.3 (0.9)	8.3 (1.0)	0.472
Hemoglobin (mg/dL)	11.4 (1.0)	11.5 (1.5)	0.890
Serum iron (micromole/l)	38.0 (24.0)	39.0 (51.0)	0.937
TIBC (micromole/l)	402.0 (118.0)	360.0 (169.0)	0.205
Ferritin (ng/mL)	765.0 (629.0)	503.0 (599.0)	0.062
Platelet count (×10^6^/microL)	168.0 (61.0)	146.0 (62.0)	0.163
PTH (ng/mL)	502.0 (627.0)	429.0 (578.0)	0.644
ProBNP (pg/mL)	163.0 (652.0)	151.0 (773.0)	0.844
LVEDD (mm/m^2^)	2.8 (0.4)	3.3 (0.4)	<0.001*
LVESD (mm/m^2^)	1.9 (0.3)	2.3 (0.4)	0.002*
LVEDV (mL/m^2^)	62.0 (14.0)	78.0 (15.0)	<0.001*
LAV index (mL/m^2^)	22.0 (11.5)	30.0 (11.5)	<0.001*
IVS (mm/m^2^)	0.9 (0.5)	1.3 (0.2)	<0.001*
GLS (%)	20.0 (3.0)	16.0 (2.5)	<0.001*
E (m/s)	65.0 (16.0)	80.0 (28.0)	0.004*
A (m/s)	70.0 (10.0)	70.0 (33.0)	0.521
E_m_ (m/s)	9.0 (2.0)	7.0 (1.5)	<0.001*
E/A	0.86 (0.3)	1.09 (0.8)	0.271
E/E_m_	7.5 (3.8)	10.6 (5.3)	<0.001*
TRPG (mmHg)	13.0 (7.0)	20.0 (15.0)	<0.001*
SPAP (mmHg)	18.0 (8.0)	30.0 (16.0)	<0.001*
TAPSE (mm/m^2^)	2.1 (0.4)	1.7 (0.3)	<0.001*

Abbreviations: dl, deciliter; EF, Ejection fraction; LVESD, left ventricular end‐systolic dysfunction; Em, early diastolic myocardial relaxation; g, gram; GFR, Glomerular filtration rate; GLS, thickness, global longitudinal strain; IVS, interventricular septal; LAV, left atrial volume index; LVEDD, left ventricular end‐diastolic dysfunction; LVEDV, left ventricular end‐diastolic volume; LVF, Left ventricular failure; M, meter; mg, milligram; min, minute; ml, milliliter; MR, mitral regurgitation; ng, nanogram; pg, pictogram. PTH, parathyroid hormone; SPAP, systolic pulmonary artery pressure; TAPSE, tricuspid annular plane systolic excursion;TIBC, Total iron binding capacity; TRPG, tricuspid regurgitation peak gradient.

* Significant difference using the Wilcoxon test.

**FIGURE 1:**
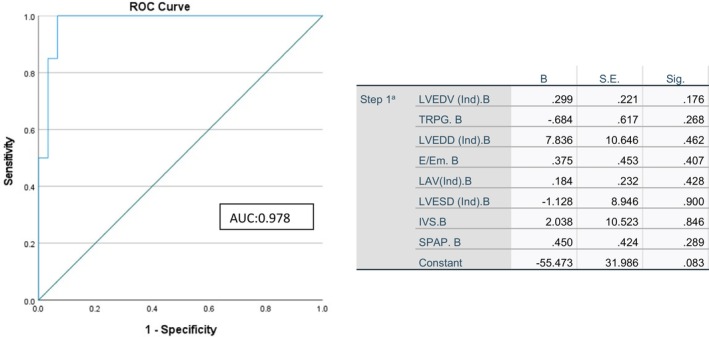
The area under the curve for echocardiographic parameters in diagnosing Left ventricular failure. The ROC curve was based on logistic multiple regression of echocardiographic parameters, which had an area under the curve (AUC) of 0.978, indicating the high accuracy of these preoperative echocardiographic parameters in predicting postoperative heart failure, although the predictive power of each echocardiographic parameter for heart failure was not significant in the presence of other parameters. E, peak velocity of early diastolic transmitral flow; Em, early diastolic myocardial relaxation; IVS, interventricular septal thickness; LAV, left atrial volume index; LVEDD, left ventricular end‐diastolic dysfunction; LVEDV, left ventricular end‐diastolic volume; LVESD, left ventricular end‐systolic dysfunction; SPAP, systolic pulmonary artery pressure; TRPG, tricuspid regurgitation peak gradient.

The ROC curve analysis based on logistic multiple regression was used to identify the predictors for LV dysfunction after renal transplantation based on preoperative echocardiographic parameters. The AUC for preoperative echocardiographic parameters (LVEDV, LVESD, LVEDD, IVS, E/Em, TRPG, SPAP, and LAV) is presented in Figure 1. These echocardiographic parameters could accurately predict postoperative heart failure in ESRD patients (AUC: 0.978). However, none of these parameters alone was statistically significant in the presence of other parameters for predicting postoperative heart failure.

## 4 DISCUSSION

Considering that one of the most important causes of morbidity and mortality in patients with renal failure is heart failure, various previous studies have shown that renal transplantation improves cardiac function (Bansal et al., 2015). Renal transplantation may reverse the physiologic cardiac changes mainly due to the improvement in uremia (Wang & Shapiro, 2019). Reduced uremia affects contraction and myocardial function independent of changes in cardiac volume hematocrit and means arterial pressure (Kaesler et al., 2020; Sucharov, 2020). On the other hand, cardiovascular disease risks still exist in post‐renal transplantation due to ESRD, dialysis, and transplantation (Rao & Coates, 2018). This study aimed to identify pre‐renal transplantation predictors for post‐renal transplantation LV failure in the short term.

The current study found that all echocardiographic parameters and MR severity were reduced after renal transplantation compared to pre‐renal transplantation. These findings were in line with the findings of previous studies (Hamidi et al., 2018; Hewing et al., 2016; Lim et al., 2020; Omrani et al., 2017). Previous studies identified improvements in cardiac function for a longer duration after renal transplantation compared to the current study (Lim et al., 2020; Omrani et al., 2017). But still, the findings of the current research were in tally with the previous studies.

Furthermore, in the current study, LVEDD, LVESD, LVEDV, LAV index, IVS, E, E/A, E/E_m_, TRPG, SPAP, and TAPSE were remarkably higher in patients with post‐renal transplantation LV failure compared to patients without post‐renal transplantation LV failure. At the same time, GLS and E_m_ were significantly lower in patients with post‐renal transplantation LV failure compared to patients without post‐renal transplantation LV failure; in a previous study, patients who died of cardiac complications, including heart failure, after renal transplantation were found to have higher LVESD, LVEDD, LA, and wall maximal thickness (Sharma et al., 2007). These findings were consistent with the findings of the present study.

This study found that pre‐renal transplantation echocardiographic parameters (LVEDV, LVESD, LVEDD, IVS, E/Em, TRPG, SPAP, and LAV) could predict post‐transplantation LV failure with high accuracy (AUC: 0.978). To our knowledge, no study has yet assessed the predictive value of pre‐renal transplantation echocardiographic parameters on LV function after renal transplantation. It was previously shown that LVESD and LVEDD were predictors of mortality due to cardiac conditions in post‐renal transplantation patients (Sharma et al., 2007). In contrast to the findings of previous studies that suggested prognostic value for NT‐proBNP on the outcome of renal transplantation, the current study found that pre‐renal transplantation NT‐proBNP was not a good predictor for post‐renal transplantation LV function (Untersteller et al., 2016).

One of the strengths of the current study was the inclusion of a comparable number of patients with and without post‐renal transplantation LV failure. This enabled the statistical comparison of study parameters between groups and identifying the predictive value for each parameter. A limitation of this study was the presence of many covariates that prevented us from performing logistic regression analysis. Therefore, it is recommended that further studies assess the relation between these parameters and LV function in a matched case–control or cohort design.

## 5 CONCLUSION

The results of this research showed that kidney transplantation should be considered in heart failure patients, because long‐term dialysis may lead to myocardial dysfunction, and kidney transplantation can lead to improved heart function in transplant patients. Also, examining echocardiographic parameters (LVEDV, LVESD, LVEDD, IVS, E/Em, TRPG, SPAP, and LAV index) together before kidney transplantation are the best predictors of LV failure after transplantation.

## AUTHOR CONTRIBUTIONS

**Hoorak Pourzand**: Conceptualization, Methodology, Supervision, Resource and investigation; **Mahboobeh Sheikhani**: Data Curation, Contact with participants, Investigation; **Mahnaz Ahmadi**: Investigation, Supervision; **Negar Morovatdar**: Formal Analysis, Software; **Sara Afshar**: Participating in data Collection, Revising manuscript; **Zahra Shahinfar**: Data curation, Investigation, Analyzing data, Writing original draft and critically Editing.

## FUNDING INFORMATION

This perusal was financially supported by grant Number: 961778 from Mashhad University of Medical Sciences.

## CONFLICT OF INTEREST STATEMENT

The authors declare that there is no conflict of interest.

## CONSENT

Written informed consent was obtained from all patients included in this study.

## Data Availability

Data are available upon sensible request.
